# A Novel Robotic Bronchoscope System for Navigation and Biopsy of Pulmonary Lesions

**DOI:** 10.34133/cbsystems.0013

**Published:** 2023-03-15

**Authors:** Xingguang Duan, Dongsheng Xie, Runtian Zhang, Xiaotian Li, Jiali Sun, Chao Qian, Xinya Song, Changsheng Li

**Affiliations:** ^1^ School of Medical Technology, Beijing Institute of Technology, Beijing 100081, China.; ^2^ School of Mechatronical Engineering, Beijing Institute of Technology, Beijing 100081, China.

## Abstract

Transbronchial biopsy sampling, as a minimally invasive method with relatively low risk, has been proved to be a promising treatment in the field of respiratory surgery. Although several robotic bronchoscopes have been developed, it remains a great challenge to balance size and flexibility, while integrating multisensors to realize navigation during complex airway networks. This paper proposes a novel robotic bronchoscope system composed by end effector with relatively small size, relevant actuation unit, and navigation system with path planning and surgical guidance capability. The main part of the end effector is machined by bidirectional groove on a nickel–titanium tube, which can realize bending, rotation, and translation 3 degrees of freedom. A prototype of the proposed robotic bronchoscope system is designed and fabricated, and its performance is tested through several experiments to verify the stiffness, flexibility, and navigation performance. The results show that the proposed system is with good environment adaptiveness, and it can become a promising biopsy method through natural cavity of the human body.

## Introduction

Lung cancer, according to GLOBOCAN, has become the second most cancer incidence and the most cancer mortality malignant tumor in the world, which takes the lives of approximately 1.8 million people in 2020 [1]. The reported 1-year survival rate for stage V is just 15% to 19% compared with 81% to 85% for stage I [2], which means that the early differentiation of benign and malignant pulmonary nodules, where the latter is lung cancer with high possibility, can effectively reduce mortality. In clinical, the percutaneous needle biopsy is a common method to perform diagnosis; however, the high risk of complications such as pneumothorax and pulmonary hemorrhage limits its wide application [3,4]. Therefore, transbronchial biopsy sampling, as a minimally invasive method with relatively low risk, has been gradually favored by surgeons and scholars in recent years [5–7].

For transbronchial biopsy sampling, one challenge is to reach the target point accurately and quickly in the complex multibranched human coelomic tract environment, highlighting the significance of navigation system [8]. The superDimension system by Medtronic Inc. and bronchoscope navigation system by LungCare Med Inc. adopted electromagnetic (EM) localization system and attached EM sensors at the end or near the end of the bronchoscope [9], which is called EM navigation bronchoscopy that is first conceptualized more than 20 years ago [10]. Nevertheless, the high practical difficulty and long learning cycle block its promotion. Lin et al. [11] introduced continuum robot and augmented reality to perform teleoperated bronchoscopy to replace the traditional manual operation, but they focus on the navigation framework, and there is no surgical tool channel due to the space limitation. Monarch platform by Auris Health and Ion Endoluminal System by Intuitive Surgical are the 2 most common worldwide robotic-assisted bronchoscopies in clinical [12,13], which has been proved to have the ability to potentially overcome some limitations of the currently available guided bronchoscopy systems and increase the diagnostic yield because of their stability, adjustable angulation, and peripheral visualization [14]. Monarch adopted EM localization system and virtual bronchoscopy, while Ion took shape reconstruction technology by fiber Bragg grating for navigation. The outer diameters of them are 4.2 and 3.5 mm, respectively, which prevents them from accessing deeper according to the inner diameter of airways with high generations [15].

The mechanism of the end effector is the key to the robotic bronchoscope moving flexibly in the airway, and it is a hot topic in the field of natural orifice transluminal surgical robots [16–19]. The most common ways are multiple interlocked segments and nickel–titanium (Ni–Ti) tube groove [20]. Liu et al. [21,22] proposed an end effector with multiple segments, which has an outer diameter of 2.2 mm while keeping a large inner lumen with a diameter of 1.44 mm for bronchoscopic instruments. Mitros et al. [23] adopted silicone tube and Ni–Ti wire to develop moving structure and tested in a 3-dimensional (3D) printed bronchus-like model to verify the effectiveness. Qi et al. [24] introduced Ni–Ti tube and conducted the finite element simulation to determine suitable slotting width. Chitalia et al. [25] designed a double segment structure with 1-way and 2-way grooves to provide with different flexibilities and stiffnesses. Coemert et al. [26] machined required working channels directly on the Ni–Ti rod and carved the grooves laterally by laser cutting, which is a good way to save space. However, as is shown in Table 1, the above methods, in varying degrees, have some limitations, such as oversized end effector, poor multisensor compatibility, and limited motion capacity in narrow luminal passages, which are not suitable for navigation and sampling deep into the bronchus.

**Table 1.** Review and comparison with existing works.

Robotic system	Navigation capability	Robotic mechanism	Size of end effector	Variable stiffness	Variable length of bending section
superDimension [9]	✓		–		
LungCare	✓		–		
Lin et. al. [11]	✓		–		
Ion [12]	✓	✓	4.2 mm		
Monarch [12]	✓	✓	3.5 mm		
Liu et. al. [21]		✓	2.2 mm		
Proposed system	✓	✓	3.3 mm	✓	✓

In this study, a novel robotic bronchoscope system is proposed to perform minimally invasive pulmonary lesions sampling. Compared with the existing works, the proposed system integrates the navigation system and robotics mechanism design to realize a relatively small size of end effector. The main part of the end effector is fabricated by bidirectional groove on a Ni–Ti tube. With additional tip section and several spacer discs, the end effector can achieve bending, rotation, and translation 3 degrees of freedom (DOFs). Moreover, the abilities of variable stiffness and variable length of bending section are also added to the mechanical design, which can effectively improve the flexibility and environment adaptiveness. The variable stiffness capability can allow the proposed robotic system to change the stiffness due to different demands at different stages of biopsy surgery, while variable length of bending section can increase the flexibility when passing through complicated bifurcated trachea. An endoscope and EM tracking system are integrated into the system as well, allowing the robot to reach the target lesion in the bronchus through the guidance of surgical path planning and navigation algorithm.

The rest of this article is organized as follows. Materials and Methods section introduces the mechanism design, working principle, and algorithms, and Experiments and Results section describes the fabrication of the prototype and several performance verification experiments. Discussion and Conclusion section analyses the contribution and limitation of the proposed system, as well as future work

## Materials and Methods

### Mechanism design

#### Robotic bronchoscope overview

**Fig. 1.**
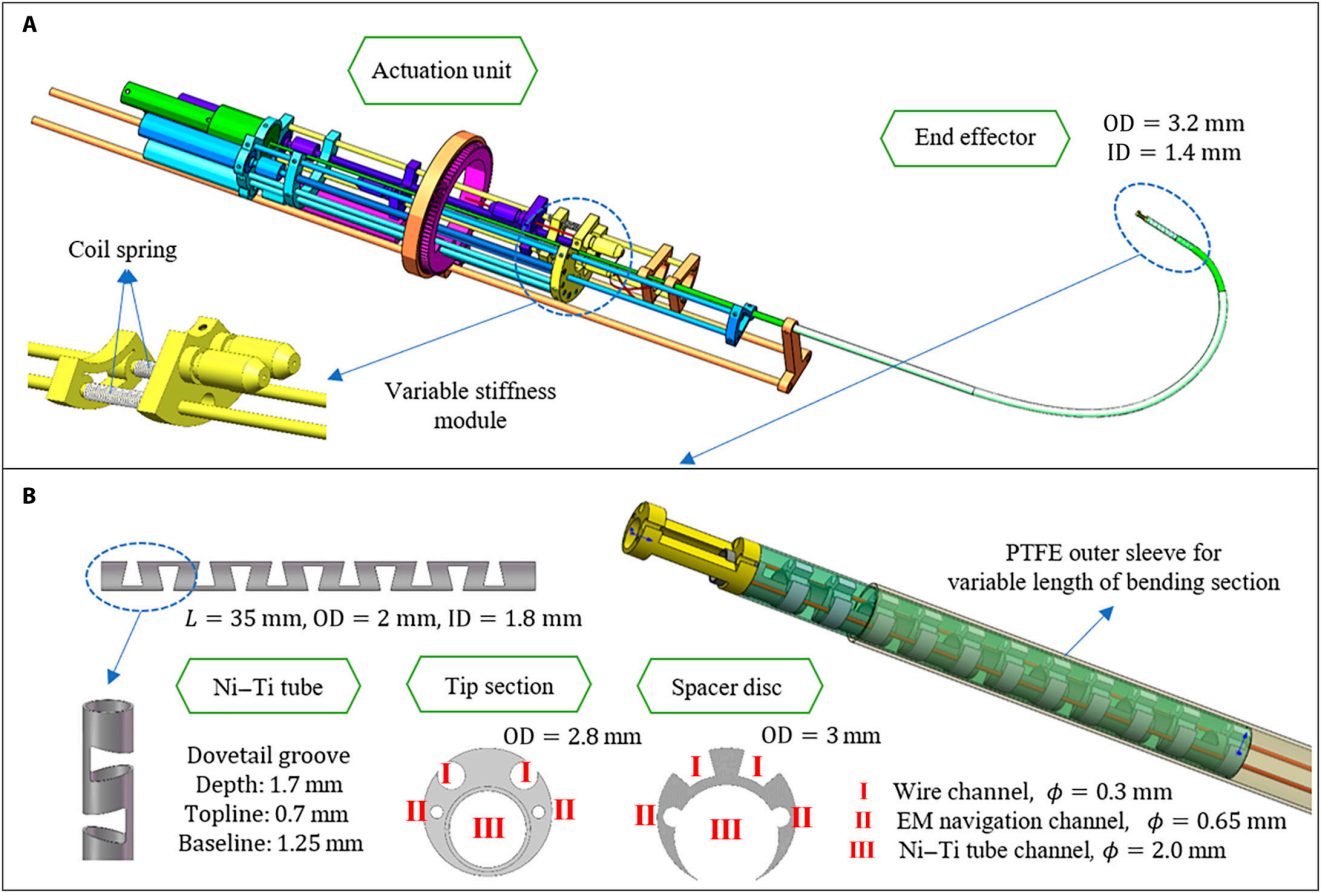
Mechanism design of the robotic bronchoscope system. (A) The overall system contains 2 parts: end effector and actuation unit. Coil springs are added to realize variable stiffness capability. OD, outer diameter; ID, inner diameter. (B) End effector is composed by Ni–Ti tube, tip section, and several spacer discs. PTFE outer sleeve is for the variable length of bending section capability.

The mechanism design begins with DOF discussion. To close to the operation mode of conventional bronchoscope, the proposed system allows the robotic bronchoscope to move forward and backward, rotate around the axis direction, and bend in radial direction, which is conducive to clinical learning and promotion. As is shown in Fig. 1A, the mechanism design can be mainly divided into 2 units: the end effector and the actuation unit.

The end effector is designed as a continuum mechanism, and the outer diameter of it is 3.2 mm, which is smaller than the outer diameter of products in commercial. For purpose of small overall size, there is only one internal working channel larger than 1.4 mm for the installation of camera module and biopsy tools, which can be easily changed according to the surgical procedure. There are also 2 EM navigation sensor channels with a diameter of 0.65 mm used for navigation system apart from the working channel. Moreover, variable length of bending section, as one of the state-of-art design in continuum robots, is also considered in the proposed robotic bronchoscope system to increase the flexibility when passing through bifurcated trachea. In practice, a polytetrafluoroethylene (PTFE) sleeve with relatively large stiffness is put on the outside, and as the sleeve moves, the length of the exposed bending section become longer or shorter, which can also increase the reachable workspace of the end effector.

Actuation unit is composed by 2 Ni–Ti wires with a diameter of 0.24 mm, a number of screw slider modules and gear transmission modules. Two coil springs is added to realize variable stiffness capability, allowing the proposed robotic system to change the stiffness due to different demands at different stages of biopsy surgery. For instance, small stiffness is needed during the process of achieving the target position under navigation, while large stiffness is used to preserve shape and position during the process of inserting biopsy needle and sampling.

#### End effector

As is shown in Fig. 1B, the end effector consists of bending section, tip section, and several spacer discs. The bending section is Ni–Ti tube with an outer diameter of 2 mm and an inner diameter of 1.8 mm, and the bending DOF is achieved by cutting groove on the Ni–Ti tube. Compared with the commonly used snake bone tube, the Ni–Ti tube is with better elasticity that increases the flexibility when passing through the complex bronchi. For example, as the robot is accessing deeper, the inner diameter of bronchi is getting smaller, and the proposed end effector can adopt a “prebent” state, which means pulling the corresponding Ni–Ti wire in the straight cavity so that the Ni–Ti tube can remain straight in the straight cavity and bend immediately once it reaches the bifurcation area with room for bending.

According to the common consensus, the width, depth, and shape of the groove will have a certain impact on the quality of the end effector. The system adopts dovetail grooves due to the requirements of relatively large bending angle and a certain position constraint. In addition, some rounded corners are added to prevent stress concentration and fracture. Through finite element analysis of the model and strength check, the ultimate stress of the model is 628 MPa, which is less than its yield strength, proving that the deformation is within its limit.

The tip section and spacer discs are designed to fix the wire outside the Ni–Ti tube to realize a relatively small size and rational utilization of space. The tip section is placed at the most front end of the integral end effector to perform anchor position for EM sensors, interior channel, and Ni–Ti wires and connect them into a whole. According to the characteristics and integration of EM navigation system, part of the cylinder of the peripheral structure is cut off to reduce the metal material and expose the EM sensors.

The spacer discs also act as wire groove and outer protect baffle to protect the Ni–Ti tube from overstretched. The outer layer is wrapped with a thin wall hose to achieve the sealing of the whole device to create a relatively dry environment for the interior structures and devices. The outer diameter of the tip section, spacer disc, and silicone hose are 3, 2.8, and 3 mm, respectively.

### Kinematic and workspace analysis

Traditional series commonly use Denavit–Hartenberg parameters method to perform kinematic analysis; however, this method does not apply to continuum robots that consist of rigid joints and connecting wires [27]. This paper adapts the method proposed by Hu et al. [28] to analyze the kinematics of the proposed robotic bronchoscope system. The analysis of this kinematic model is based on the following prerequisites.

• Simplified the bending section as a special manipulator with a single joint and one DOF.
• Each joint of the bending section is assumed to be a smooth continuous curve with equal curvature during the process of end effector bending.
• The mass of tip section and spacer discs are relatively quite small, so the influence of gravity on the end effector can be ignored and only the influence of rope tension is taken into consideration.

On the basis of the aforementioned model concept, the kinematics can be decomposed into 2 mappings. First is “Actuator space—Configuration space mapping” and the other is the “Configuration space—Task space mapping”. The “Actuator space—Configuration space mapping” is to establish the relationship between rope motion and the bending configuration, while the “Configuration space—Task space mapping” is to compute the tip position and orientation based on the bending configuration.

**Fig. 2.**
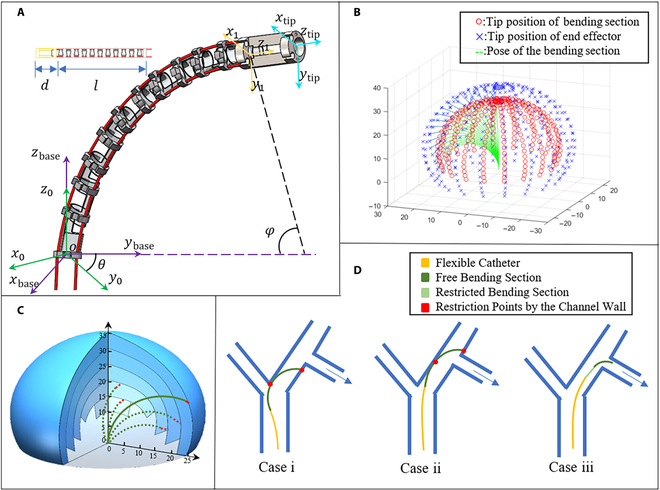
Kinematics and cavity passage capability analysis. (A) Kinematics model and coordinate systems. (B) Regular reachable workspace. (C) Augmented workspace by variable length of bending section capability. (D) Examples of robot posture in bronchus. Case i and ii, restricted by bifurcation and channel wall without certain capability; case iii, successful entry into target bronchus with certain capability.

The end of the continuum, as is presented in Fig. 2A, can be divided into bendable part with length *l* and rigid inflexible part (the tip section) with length *d*; the reference coordinate system is on the base of the end effector.

According to screw theory, the screw ***ν*** and exponential product of the first rotation joint can be written as:

$$\nu =\left[\begin{array}{c} -\omega \times q\\ \omega \end{array}\right]$$ (1)

$$e^{\left[\nu \right]\theta }=\left[\begin{array}{cc} e^{\left[\omega \right]\theta } & \left(I-e^{\left[\omega \right]\theta }q\right)\\ 0 & 1 \end{array}\right]$$ (2)

where *θ*, ***ω***, and ***q*** denote rotation angle, rotation vector, and the coordinate of a point on the rotation axis, respectively, and then the transform matrix from $T_{\text{base}}^{0}$ can be gotten by:

$$T_{\mathrm{base}}^{0}=e^{\left[\nu \right]\theta }=\left[\begin{array}{cccc} \cos\theta & -\sin\theta & 0 & 0\\ \sin\theta & \cos\theta & 0 & 0\\ 0 & 0 & 1 & 0\\ 0 & 0 & 0 & 1 \end{array}\right].$$ (3)

When the bending section is moving, the transform matrix $T_{0}^{1}$ can be decomposed to rotation and translation in the bending plane, which can be described as:

$$\begin{array}{c} \begin{array}{c} T_{0}^{1}=\text{Trans}\left(0,,,\frac{l}{\phi }\left(1-\cos\phi \right),,,\frac{l}{\phi }\sin\phi \right)\times \mathrm{Rot}\left(x,,,\phi \right)\\ =\left[\begin{array}{cccc} 1 & 0 & 0 & 0\\ 0 & \cos\phi & -\sin\phi & \frac{l}{\phi }\left(1-\cos\phi \right)\\ 0 & \sin\phi & \cos\phi & \frac{l}{\phi }\sin\phi \\ 0 & 0 & 0 & 1 \end{array}\right]. \end{array} \end{array}$$ (4)

For the rigid unbendable part with length *d*, the transform matrix $T_{1}^{\mathrm{tip}}$ can be written as:

$$T_{1}^{\mathrm{tip}}=\left[\begin{array}{cccc} 1 & 0 & 0 & 0\\ 0 & 1 & 0 & 0\\ 0 & 0 & 1 & d\\ 0 & 0 & 0 & 1 \end{array}\right].$$ (5)

Then, the mapping from configuration space to task space can be calculated by:

$$\begin{array}{c} T_{\mathrm{base}}^{\mathrm{tip}}=T_{\mathrm{base}}^{0}T_{0}^{1}T_{1}^{\mathrm{tip}}=\left[\begin{array}{cccc} \cos\theta & -\cos\phi \sin\theta & -\sin\phi \sin\theta & \frac{l}{\phi }\sin\theta \left(\cos\phi -1\right)-d\cdot \sin\phi \sin\theta \\ \sin\theta & \cos\phi \cos\theta & \sin\phi \cos\theta & -\frac{l}{\phi }\cos\theta \left(\cos\phi -1\right)+d\cdot \sin\phi \cos\theta \\ 0 & \sin\phi & \cos\phi & \frac{l}{\phi }\sin\phi +d\cdot \cos\phi \\ 0 & 0 & 0 & 1 \end{array}\right]. \end{array}$$ (6)

The mapping from actuator space to configuration space is relationship between Δ*l* and *ϕ*, which can be figured out by:

$$\Delta l=l_{\text{post}}-l_{\mathrm{pre}}=\rho_{\text{post}}\phi -\rho_{\mathrm{pre}}\phi =\phi \left(\rho_{\text{post}}-\rho_{\mathrm{pre}}\right)=\phi r$$ (7)

$$\phi =\frac{\Delta l}{r}$$ (8)

where *l*_post_, *ρ*_post_, *l*_pre_, and *ρ*_pre_ denote the length and the curvature of the wire after and before bending, respectively.

The workspace of the proposed end effector can be computed by the model and formula above with constant parameters *l*, *d*, and *r* and variable parameters *θ* and Δ*l*. The analysis is conducted via MATLAB and the specific range is:

$$\left\{\begin{array}{ll} & l=29\;\mathrm{mm}\\ & d=6\;\mathrm{mm}\\ & r=1.2\;\mathrm{mm}\\ & \Delta l\leq 3.5\;\mathrm{mm}\\ & \theta =\left[0,2\pi \right]. \end{array}\right.$$ (9)

Figure 2B shows the regular workspace of proposed end effector, which is the area of that the tip can reach. Blue crosses and red dots illustrate the tip of whole end effector and bending section, while green curve shows the shape of the bending section. It can be easily observed that the workspace is a space close to a spherical shell when the robot is at a certain point, which means that the interior positions cannot be reached and the translation of whole robot is needed to combine to realize. Under such circumstances, the robot’s ability to pass through the multibranched cavity will be limited, and it may easily get restricted by channel wall (see cases i and ii in Fig. 2D). Therefore, the idea of variable length of bending section is introduced in the proposed system, which means the value of the length *l* and the upper bound of the variable parameter Δ*l* can be altered in theoretical. Figure 2C shows that the augmented workspace and most spatial positions inside the sphere become reachable, which effectively solve the above problems (see case iii in Fig. 2D).

### Navigation system

#### System framework

Traditional bronchoscopy relies on the surgeons to localize the tip of bronchoscope by observing the endoscopic image combined with preoperative 3D image, which is highly dependent on the experience and competence. Hence, for years, many researchers studied on the navigation methods and proposed various approaches, such as 2D/3D registration, image characteristics recognition, and EM image guidance [29–31]. In this study, a navigation system integrated both endoscope and EM tracking system is adopted to orient and guide the flexible robotic bronchoscope inside the complex airway networks. The hardware components mainly include EM tracking system and endoscope module.

For the preoperative stage, the system first acquires the 3D airway model and location of target lesions by the computed tomography data, and then surgical planning algorithm automatically calculates the internal path after choosing the entry point and target point manually. For the intraoperative stage, the key procedure is to obtain the position of patient in the EM tracking system and deduce the relative position from the patient to the robot.

**Fig. 3.**
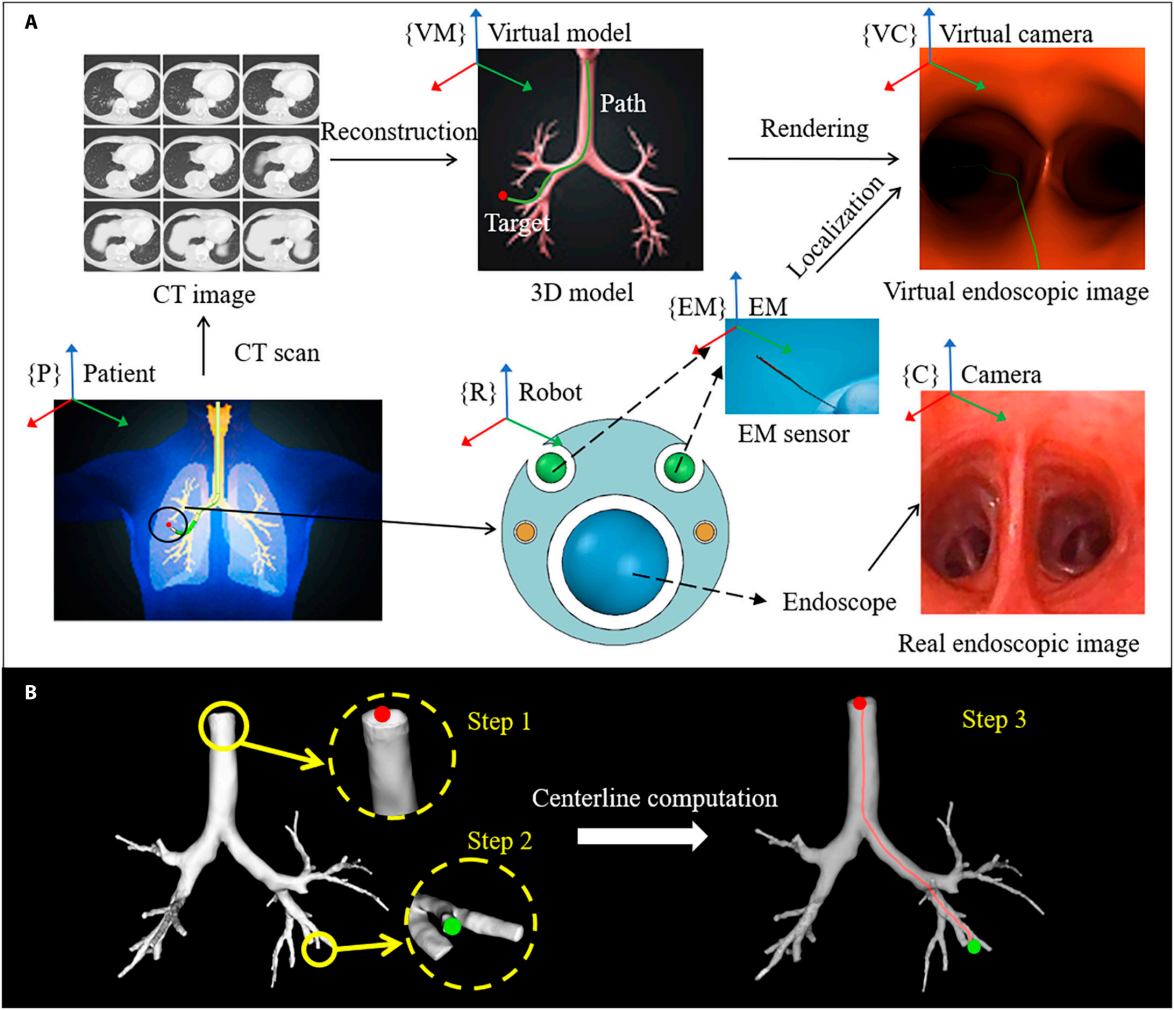
Navigation system workflow. (A) The overall framework and the purpose of it are to localize the tip of robotic bronchoscope in the complex airway networks. (B) Surgical path planning procedures. Step 1 is the entry point, step 2 is the target point, and step 3 is the generated path. CT, computed tomography.

Denote the reference frame of patient, virtual model, virtual camera, robot, EM system, and camera as {P}, {VM}, {VC}, {R}, {EM}, and {C} respectively (see Fig. 3A). The purpose of the navigation system is to obtain the transform matrix from patient to robot $T_{P}^{R}$, which is calculated by:

$$T_{P}^{R}=T_{P}^{C}T_{C}^{R}$$ (10)

where $T_{P}^{C}$ is the transform matrix from patient to camera, $T_{C}^{R}$ is the transform matrix from camera to robot. According to the mechanism design of the end effector, $T_{C}^{R}$ can be gotten by:

$$T_{C}^{R}=T_{C}^{\mathrm{EM}}{\left(T_{R}^{\mathrm{EM}}\right)}^{-1}$$ (11)

where $T_{C}^{\mathrm{EM}}$ is the transform matrix from EM to camera and $T_{R}^{\mathrm{EM}}$ is the transform matrix from EM to robot, which can be easily gotten by their given relationship. For $T_{P}^{C}$, the transform of the virtual camera to virtual model ($T_{\mathrm{VM}}^{\mathrm{VC}}$) can be gotten by the transform of the robot to the patient ($T_{P}^{C}$) when the virtual image is matched to the real image, which is a given assumption that has been widely used in thecorresponding field, and the relationship is:

$$T_{P}^{C}=T_{W}T_{\mathrm{VM}}^{\mathrm{VC}}$$ (12)

where ***T****_W_* is the transform matrix from real world to virtual world that can be obtained by a simple and fast calibration process. In this case, it is equal to $T_{\mathrm{EM}}^{\mathrm{VM}}$, since EM and VM perform as the reference frame for real world and virtual world. Finally, substitute Eqs. 11 and 12 into Eq. 10,

$$T_{P}^{R}=T_{\mathrm{EM}}^{\mathrm{VM}}T_{\mathrm{VM}}^{\mathrm{VC}}T_{C}^{\mathrm{EM}}{\left(T_{R}^{\mathrm{EM}}\right)}^{-1}.$$ (13)

#### Surgical path planning

Surgical path planning always plays an important role in the navigation system, and it is also the case in the robotic bronchoscopy system. To do this, automatic path generation from 3D model is adapted from computational geometric description method of blood vessel [32]. For tubular structures, the typical representation is centerline, which can be defined as the line drawn from the 2 outermost sections of a tubular structure that locally maximizes the distance from boundary. It cannot only provide visual guidance in virtual endoscopy navigation but also provide help for robot control in the future.

In implementation, first, an approximation of the medial axis of the models, the embedded Voronoi diagram, is computed [33]. Then, the Eikonal equation is solved on the Voronoi diagram by means of fast marching method [34]. From the given definition of centerlines, it can be deduced that centerlines must lie on the medial axis of the tubular surface. Therefore, the centerline calculation problem is formulated in terms of energy minimization, which can be written as:

$$E_{\text{Certerline}}\left(C\right)=\int_{0=C^{-1}\left(p_{0}\right)}^{L=C^{-1}\left(p_{1}\right)}F\left(C\left(s\right)\right)\mathrm{ds}$$ (14)

where ***C*** = ***C***(*s*) (*s* being arc length) is the centerline and ***p***_0_ and ***p***_1_ denote the entry point and target point in Fig. 3B, *F*(***x***) is a scalar field that relates to values of Voronoi diagram as in [35]. Centerlines can be viewed as deformable lines linked between 2 fixed points and finding the stable configuration at a minimum of $E_{\text{Certerline}}$, which is equivalent to solving the following evolutionary Euler–Lagrange equation.

$$\frac{dC}{\mathrm{dt}}=-\nabla F\left(x\right)$$ (15)

The numerical solution is also approximated by fast marching method mentioned above, and after that, an ideal surgical path is generated to act as the guideline during the operation. The path that figured out by the algorithm will be rendered into the virtual endoscopic view, aiming to provide visual guidance for the surgeons, and in the future, it can also be integrated into the control unit to realize automatic marching in the airway networks.

## Experiments and Results

### Stiffness characterization

**Fig. 4.**
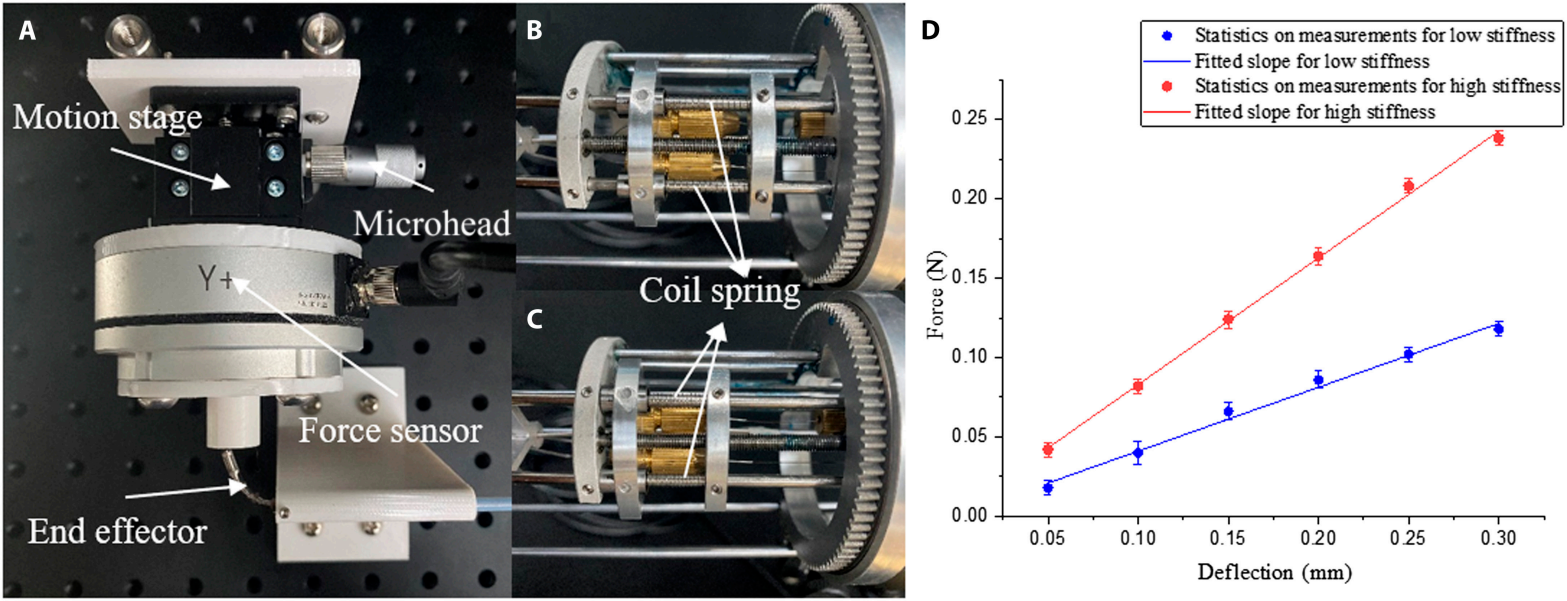
Stiffness test. (A) Experimental setup. (B and C) Setup for low and high stiffness states. The tensions of the coil springs are different. (D) Results of stiffness test.

The stiffness characterization of the prototype is evaluated by applying deflections at the tip of the end effector and measuring the reaction force [36]. The experimental setup is shown in Fig. 4A. A force sensor (KWR75A, Kunwei Technology Ltd.) is carried by a motion stage. The motion stage has one DOF, which is in line with the given measurement direction. The driven rope relaxation state is a state with quite small stiffness, which allows the end effector the best environmental adaptability. Therefore, the stiffness test is carried out at the bending state. The end effector is set under 2 configurations separately, including low and high stiffness, which is realized by different tensions of coil springs of variable stiffness module (see Fig. 4B and C).

During the experiment, the end effector is bent in the measurement direction, and the motion stage is moved to a position that just touching the tip. Then, the microhead is rotated to move the motion stage at a step of 0.05 mm. The reaction force is recorded by the force sensor readings. The test is repeated 5 times for each state of stiffness, and the results are shown in Fig. 4D. The stiffness is estimated by curve fitting tool in Origin software. The polynomial fitting is chosen as the fitting method, and result shows a linear relationship between force and deflection with an *R*^2^ value higher than 0.98. It is obvious that there is a clear difference in the slopes of different stiffness states.

### Flexibility characterization

**Fig. 5.**
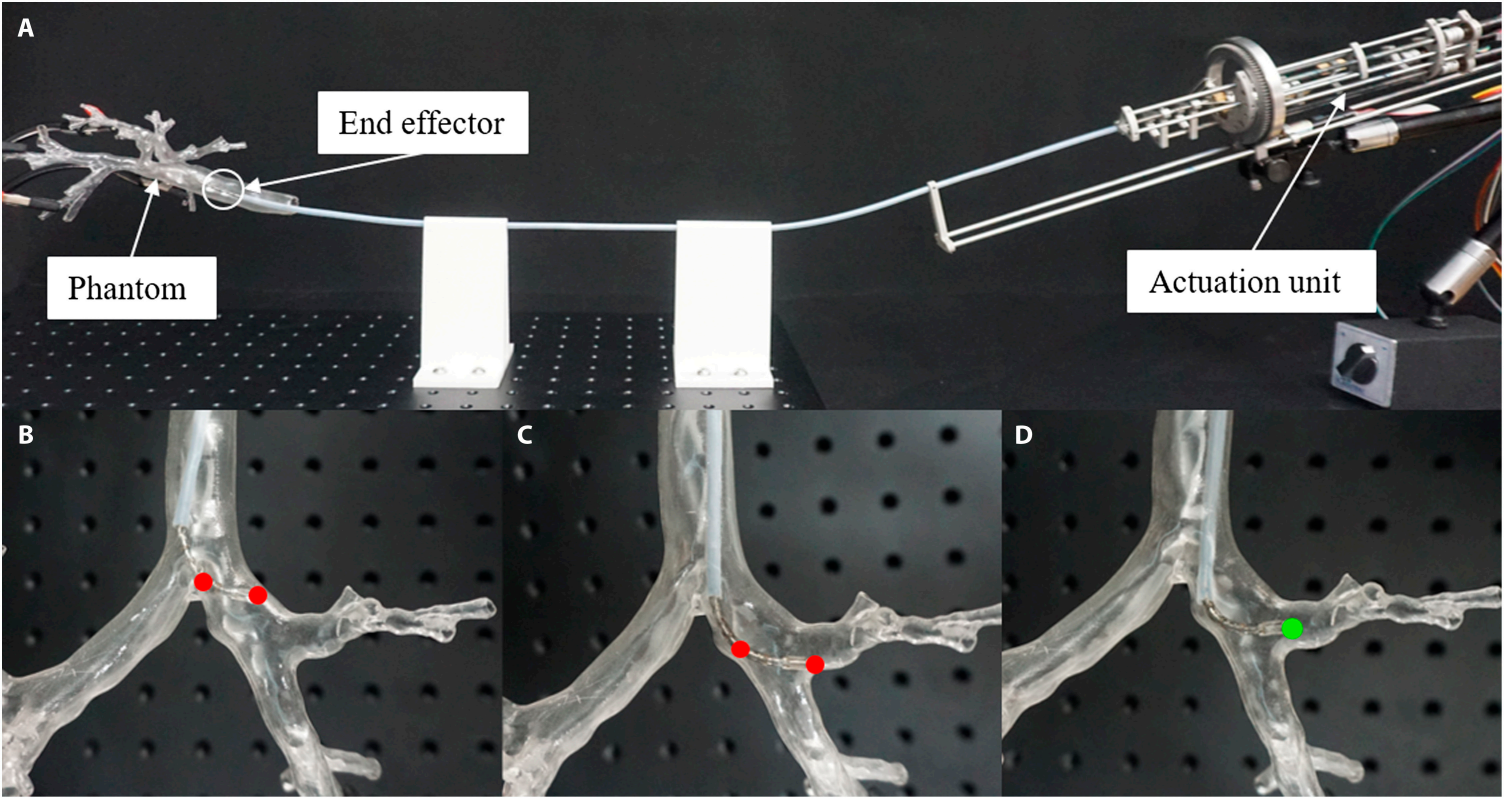
Flexibility test. (A) Experimental setup. (B to D) Examples of robot posture in phantom. Red points illustrate that the robot is restricted by channel wall, while green point illustrates that the robot enters target branch successfully with a relatively short length of bending section.

The flexibility characterization is tested in a phantom, and the experimental setup is illustrated in Fig. 5A. All DOFs including the movement of PTFE sleeve are actuated by brush DC motors (RE13, Maxon Motor Inc.), and each motor is connected with a motor driver (RMDS-305, RoboModule Technology Ltd.). The drivers communicate with a PC via the CAN Bus protocol. A CAN-to-USB interface (USBCAN-II C, Guangcheng Technology Ltd.) is used to connect the motor drivers to the PC. The motors are connected to lead screws that convert the power generated by the motor into feed velocity for pulling/pushing the sliders. The Ni–Ti wires are fixed on the sliders corresponded to bending DOF by the bit clip so that they can be pulled/pushed along the lead screw.

The specific parameters of the fabricated prototype are as shown in Table 2, and for the pulmonary lesions sampling process, the patient firstly needs to adjust to an appropriate posture. After the confirmation by the primary surgeon, the image capture system is deployed, and the surgeon manually placed the actuator at the appropriate position in the main airway. According to the results of the image capture system and positioning tracking system, combined with the preoperative medical image diagnosis, real-time visual guidance is provided and displayed in the display system. Then, the surgeon controls the actuator to reach the target nodule position under the visual guidance. Pressure is applied to the syringe to inflate the sacculus aeris and hold in position, and the surgeon can replace the image capture system with a biopsy sampling tool through the tool channel. Exhaust the sacculus aeris and remove the actuator after several puncture process. Such process is demonstrated in Movie S1.

**Table 2.** Main parameters of the proposed robotic system.

Parameters	Value	Units
DOF	3	–
Diameter of the end effector	3.3	mm
Diameter of the tool channel	1.4	mm
Length of insertion part	700	mm
Maximum bending angle	150	degrees
Maximum rotation angle	360	degrees
Operating mode	Master-slave teleoperation	–

The phantom is made by 3D printing technology, and the 3D digital airway model is obtained by several medical image processing methods such as segmentation, reconstruction, and optimization (using Mimics Medical and Geomagic Wrap software). The data are actual clinical data from an open-source lung cancer dataset called non-small-cell lung cancer radiogenomics [37]. The end effector is first placed at the main airway of the phantom, and then translation, bending, and rotation DOFs are used alternately to allow the robotic system moving inside the phantom and reaching different branches (see Movie S1 for detailed demonstration). Variable length of bending section capacity is also used when necessary during the test. In some cases, such as passing through a series of bifurcates with a relatively large angle, as is shown in Fig. 5B and C, the robot will easily be restricted by the channel wall with a long bending section. However, this problem can be effectively solved after changing the length of bending section (see Fig. 5D), which demonstrates that such function can improve the flexibility and environment adaptiveness of the robotic bronchoscope system and have certain clinical application value (see Movie S2 for detailed demonstration).

### Navigation-assisted intervention experiment

**Fig. 6.**
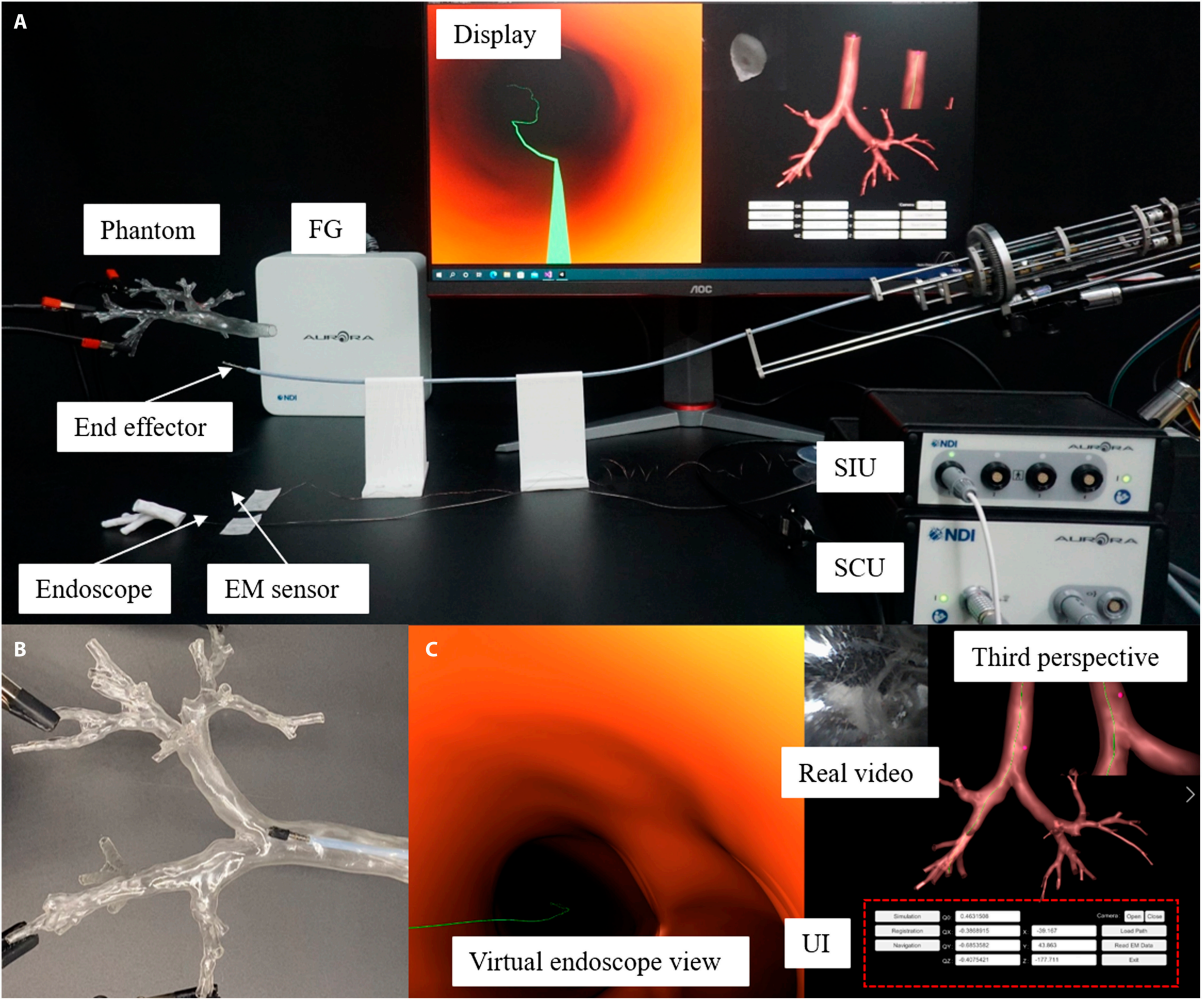
Navigation-assisted intervention experiment. (A) Experimental setup. (B) One scene of end effector in the phantom during experiments. (C) Corresponding navigation system display. FG, field generator; SCU, system control unit; SIU, sensor interface unit; UI, user interface.

To evaluate the navigation system’s performance in a real application, an intervention experiment is conducted. As is illustrated in Fig. 6, the purpose of this experiment is to verify the match between the displayed position of the virtual endoscope system and the actual position of the end effector in the phantom. Meanwhile, the virtual endoscope view and the real video from actual endoscope module should also match with each other.

The experimental setup is shown in Fig. 6A. The navigation interface and image rendering is realized by Unity3D software. An endoscope module and a 6-DOF EM sensor (Aurora, Northern Digital Inc.) are assembled on the tip section of proposed end effector to provide vision and position information. The endoscope module, with an outer diameter of 1 mm, is integrated by an image sensor (OVM 6948, OmniVision Inc.) with a light-emitting diode module. The 6-DOF EM sensor is used to replace the two 5-DOF sensors in the design for the convenience of assembly and protection of fragile tiny sensors. Field generator, system control unit, and sensor interface unit of the EM tracking system are also included, and other settings are consistent with flexibility characterization test.

The intervention experiment has demonstrated the feasibility of the proposed robotic bronchoscope navigation system (see Fig. 6B and C). During the experiment, the position displayed by the third perspective of virtual endoscope is consistent with the position of the end effector’s tip relative to the airway phantom from visual observation. The virtual endoscope view also matches with the real video acquired by endoscope module as expected (see Movie S3 for detailed demonstration).

## Discussion and Conclusion

This paper presents a novel robotic bronchoscope system aiming to assist navigation and sampling for pulmonary lesions. Compared with the existing works, this study focuses on the integration of whole systems to provide navigation and assistance during biopsy process. The system consists of end effector with relatively small size, relevant actuation unit, and navigation system with path planning and surgical guidance capability. The main part of the end effector is machined by bidirectional groove on a Ni–Ti tube, which is with great elasticity that allows the robotic system moving flexibly in complex luminal environment. The variable stiffness and variable length of bending section are also added to the mechanical design to further improve the flexibility and environment adaptiveness. Kinematics and reachable workspace are analyzed to theoretically discuss about the applicable ambient of the proposed robotic system. Navigation system integrated endoscope module and EM tracking system are also introduced in this system, with surgical path planning algorithm to realize intraoperative visual guidance.

A prototype of the proposed robotic system is fabricated and verified through adequate experiments. For stiffness test, 2 typical states with low and high stiffness, respectively, is selected to test by applying deflections at the tip of the end effector and measuring the reaction force. The results demonstrate that there is a linear relationship between force and deflection and the slope for high stiffness is about 2 times of the slope for low stiffness. Hence, the proposed system can adjust its stiffness according to the demands of different surgical processes. For flexibility test, the bending, rotation, and translation 3 DOFs are tested in free space and in airway phantom. Variable length of bending section can offer the robot a more flexibly movement and better environmental adaptiveness since the robot, without such capability during the test, is often restricted by the channel wall. For navigation performance test, the results show that navigation system can offer effective guidance in the complex airway networks. As we all know, the real human will breathe when you examine with the robotic bronchoscope system. Therefore, the real scenario is dynamic and not the static phantom. This problem has also been taken into consideration in the proposed system. The solution can be divided into 2 parts, the dynamic lung model reconstruction and temporal–spatial registration method. The former one can be solved by 4D imaging method [38], while the latter one can be figured out by respiratory-gating method to align the breath phase [39], and both results compatible with the software of the proposed robotic system.

Although the system has achieved promising bronchoscopy performance, as a medical device, there are still some issues that limit the clinical application and promotion. First, the current operation is to control the motor directly, which is not convenient for the surgeons to learn and use. Second, the image sensor used in endoscope module is with low resolution due to the space limitation, making the actual endoscopic image a little vague. Third, for the calibration process of navigation system, the user needs to initially match the virtual and actual endoscopic images by vision observation. A more intelligent strategy is required.

Future work will be focused on the following aspects: (a) The mechanism will be improved including minimizing the diameter of the end effector and adopting replaceable module for diverse surgical requirements. (b) Two or more DOFs will be considered to joint control to allow the robot a more flexible movement in the airway. Joystick-based or touchless teleoperation such as gesture-based control will be introduced as a user-friendly interaction mode. (c) Image processing and computer vision algorithm will be adapted to realize an automatic and intelligent calibration method.

## Data Availability

The data used to support the findings of this study are included within the article.

## Supplementary Materials

Supplementary 1 Movie S1. Basic motor function. This video demonstrates the bending, translation, and rotation 3 DOFs in free space, and all DOFs are alternately used to allow the robotic system reaching the target branch. Moreover, the robotic biopsy process demonstration is involved, including the use of inflatable sacculus aeris and biopsy tools. Movie S2. Flexibility characterization. This video demonstrates the capability of variable length of bending section in free space and in phantom. The robot will easily be restricted by the channel wall with a long bending section, while this problem can be effectively solved after changing the length of bending section accordingly. Movie S3. Navigation-assisted intervention experiment. This video demonstrates the navigation system’s performance. The position displayed by the third perspective of virtual endoscope is consistent with the position of the end effector’s tip relative to the airway phantom. The virtual endoscope view also matches with the real video acquired by endoscope module as expected.
